# Inhibition of Histone Deacetylases 1 and 6 Enhances Cytarabine-Induced Apoptosis in Pediatric Acute Myeloid Leukemia Cells

**DOI:** 10.1371/journal.pone.0017138

**Published:** 2011-02-16

**Authors:** Xuelian Xu, Chengzhi Xie, Holly Edwards, Hui Zhou, Steven A. Buck, Yubin Ge

**Affiliations:** 1 Developmental Therapeutics Program, Barbara Ann Karmanos Cancer Institute, Wayne State University School of Medicine, Detroit, Michigan, United States of America; 2 Department of Oncology, Wayne State University School of Medicine, Detroit, Michigan, United States of America; 3 The State Engineering Laboratory of AIDS Vaccine, College of Life Science, Jilin University, Changchun, People's Republic of China; 4 Division of Pediatric Hematology/Oncology, Children's Hospital of Michigan, Detroit, Michigan, United States of America; 5 Department of Pediatrics, Wayne State University School of Medicine, Detroit, Michigan, United States of America; University of Windsor, Canada

## Abstract

**Background:**

Pediatric acute myeloid leukemia (AML) remains a challenging disease to treat even with intensified cytarabine-based chemotherapy. Histone deacetylases (HDACs) have been reported to be promising therapeutic targets for treating AML. However, HDAC family members that are involved in chemotherapy sensitivities remain unknown. In this study, we sought to identify members of the HDAC family that are involved in cytarabine sensitivities, and to select the optimal HDACI that is most efficacious when combined with cytarabine for treating children with AML.

**Methodology:**

Expression profiles of classes I, II, and IV HDACs in 4 pediatric AML cell lines were determined by Western blotting. Inhibition of class I HDACs by different HDACIs was measured post immnunoprecipitation. Individual down-regulation of HDACs in pediatric AML cells was performed with lentiviral shRNA. The effects of cytarabine and HDACIs on apoptosis were determined by flow cytometry analysis.

**Results:**

Treatments with structurally diverse HDACIs and HDAC shRNA knockdown experiments revealed that down-regulation of both HDACs 1 and 6 is critical in enhancing cytarabine-induced apoptosis in pediatric AML, at least partly mediated by Bim. However, down-regulation of HDAC2 may negatively impact cytarabine sensitivities in the disease. At clinically achievable concentrations, HDACIs that simultaneously inhibited both HDACs 1 and 6 showed the best anti-leukemic activities and significantly enhanced cytarabine-induced apoptosis.

**Conclusion:**

Our results further confirm that HDACs are *bona fide* therapeutic targets for treating pediatric AML and suggest that pan-HDACIs may be more beneficial than isoform-specific drugs.

## Introduction

Acute myeloid leukemia (AML) accounts for one-fourth of acute leukemia in children, but is responsible for more than half of the leukemia deaths in this patient population [1]. Resistance to cytarabine (ara-C)-based chemotherapy is a major cause of treatment failure in this disease [2], [3]. Therefore, new therapies for children with AML are urgently needed. Among the newer antileukemic agents that have been recently investigated in high-risk adult AML, histone deacetylase (HDAC) inhibitors [HDACIs, e.g., valproic acid (VPA) and Vorinostat (SAHA)] are particularly notable [4], [5]. The ability of HDACIs to induce cell differentiation, cell cycle arrest, and apoptosis in human leukemic cells, but not in normal cells [6], has stimulated significant interest in their potential as anti-leukemia agents. Numerous HDACIs have been developed during the last decade and the majority of these are being studied in solid tumor and hematological malignancy clinical trials, including the novel class I-selective HDACIs, MS-275 and MGCD0103, and pan-HDACIs, LBH-589 and PXD101 [4], [5].

Despite the well-characterized molecular and cellular effects of HDACIs, single-agent activity of this class of drugs has been modest. Therefore, there is an urgent need for developing rationally designed drug combinations including HDACIs. In our previous study [7], we hypothesized that VPA synergizes with cytarabine, resulting in enhanced antileukemic activities in AML cells, by inducing apoptosis. We previously examined the impact of VPA on cytarabine cytotoxicities in 4 pediatric AML cell lines and 9 diagnostic blast samples from children with *de novo* AML and demonstrated highly synergistic antileukemic activities of combined cytarabine/VPA in all of the cell lines and diagnostic blast samples, especially those with t(8;21). Our mechanistic studies revealed that cooperative induction of DNA damage by the two agents and induction of Bim by VPA underlay the observed synergistic antileukemic activities of this drug combination. Indeed, our results strongly suggested that HDACs are promising therapeutic targets for pediatric AML, however, which of the HDAC family members are involved in the synergy between cytarabine and VPA is not clear.

HDACs comprise a large group of proteins divided into four classes based on their homologies to yeast HDACs, their subcellular localizations and their enzymatic activities [8], [9]. Class I HDACs comprise HDACs 1, 2, 3 and 8 and are all homologues of the yeast rpd3 protein. They are ubiquitously expressed and are located primarily in the nucleus [8], [9]. Class II enzymes comprise HDACs 4, 5, 6, 7, 9 and 10, which are homologues of the yeast hda1 protein. These enzymes generally exhibit tissue-specific expression and shuttle between the cytoplasm and nucleus in response to cellular signals [10]. Since HDACs 6 and 10 contain two catalytic sites, these enzymes are sometimes designated as a separate subclass (Class IIb) [11]. Class III HDACs are comprised of the seven sirtuins (SIRT1-7), homologues of the yeast SIR2 protein [12]. HDAC11 contains conserved residues that are shared by both class I and class II enzymes and is classified as a class IV enzyme [13], [14].

HDACs control gene expression through chromatin modification. Recent studies have shown that exposure to HDACIs “resensitizes” AML cells to signals for differentiation and/or apoptosis, making HDACIs particularly promising agents for AML therapy [4], [5], [15]. Knockout and siRNA knockdown experiments have suggested that class I HDACs are essential for cancer cell proliferation and survival, in contrast to class II HDACs 4 and 7 [16], [17]. However, inhibition of the class II HDAC6 leads to acetylation and disruption of the chaperone function of heat-shock 90 (Hsp90) in leukemic cells [18]. Thus, although it is increasingly apparent that the class I HDAC enzymes are clinically relevant for cancer, this is less established for the class II enzymes.

In this study, we used 4 pediatric AML cell lines to identify HDAC family members which are involved in cytarabine sensitivities, and to select the optimal HDACIs that were most efficacious against pediatric AML when combined with cytarabine. We demonstrated that HDACs 1 and 6 are critical for cytarabine-induced apoptosis and suggest that pan-HDACIs, which simultaneously inhibit HDACs 1 and 6, may have the greatest potential for enhancing cytarabine activities in pediatric AMLs. Our results further support the use of HDACIs in the treatment of childhood AML.

## Materials and Methods

### Drugs

PXD101, LBH-589 and MGCD0103 were purchased from Selleck Chemicals (Houston, TX). SAHA and MS-275 were purchased from United States Biological (Swampscott, MA), and from ChemieTek (Indianapolis, IN), respectively. Cytarabine (cytosine arabinoside, ara-C) and valproic acid (VPA) were purchased from Sigma Chemical Company (St Louis, MO).

### Cell culture

The THP-1, Kasumi-1, and MV4-11 pediatric AML cell lines were obtained from American Type Culture Collection (ATCC; Manassas, VA). The CMS pediatric AML cell line was a gift from Dr A. Fuse (National Institute of Infectious Diseases, Tokyo, Japan). The parental and the engineered sublines were cultured in RPMI 1640 with 10-20% heat-inactivated fetal bovine serum (FBS; Hyclone Labs, Logan, UT) and 2 mM L-glutamine plus 100 U/mL penicillin and 100 µg/mL streptomycin in a 37°C humidified atmosphere containing 5% CO_2_/95% air.

### Enzymatic Assays of Class I HDACs Following Immunoprecipitation (IP)

THP-1 cells were treated with various concentrations of HDACIs for up to 48 h and lysed in Cell Lysis Buffer [20 mmol/L Tris-HCl (pH 8), 0.15 mol/L NaCl, 10% glycerol, and 0.5% NP40] on ice for 2 hours. After centrifugation (12,000× g for 15 minutes), 500 µg supernatant fraction (cell lysate) was incubated with 2 µg rabbit IgG, anti-HDAC1, anti-HDAC3 (Bethyl Labs, Montgomery, TX), anti-HDAC2 (CycLex, Nagano, Japan) or 1000 µg supernatant fraction was incubated with 2 µg anti-HDAC8 (Santa Cruz Biotechnology, California) overnight at 4°C, followed by incubation with 30 µL of Protein A/G® Dynabeads (Invitrogen Dynal AS, Oslo, Norway) for 3 hours at 4°C. The beads were washed three times with ice cold PBS and resuspended in HDAC Assay Buffer [40 µL; 20 mmol/L Tris-HCl (pH 8), 125 mmol/L NaCl, and 1% glycerol] for measuring HDAC enzymatic activities using the CycLex® HDACs Deacetylase Fluorometric Assay kit (CycLex, Nagano, Japan), or heated at 95°C for 5 min in 30 µl loading buffer for Western blotting.

### Western Blot Analysis

Soluble protein extracts prepared by sonication in hypotonic buffer (10 mM Tris-Cl, pH 7.0), containing 1% SDS and proteolytic inhibitors, or immunoprecipitated proteins were subjected to SDS-polyacrylamide gel electrophoresis. Separated proteins were electrophoretically transferred to polyvinylidene difluoride (PVDF) membranes (Thermo Fisher Inc., Rockford, IL) and immunoblotted with anti-HDAC1 (#2062), -HDAC2 (#2540), -HDAC3 (#2632), -HDAC4 (#2072), -HDAC5 (#2082), -HDAC7 (#2882) (Cell Signalling Technology, Beverly, MA), -HDAC6 (sc-11420, Santa Cruz Biotechnology, Santa Cruz, CA), -HDAC8 (H6412), -HDAC10 (H3412), -HDAC11 (H4539), –acetyl (ac)-tubulin (T7451) (Sigma, Saint Louis, Missouri), -HDAC9 (SH030228P, ABGENT, San Diego, CA), -ac-Histone H4, -ac-Histone H3, -ac-Histone H4, -Histone H4, or –beta-actin antibodies (Upstate Biotechnology, Lake Placid, NY), as described previously [19], [20]. Immunoreactive proteins were visualized with the Odyssey Infrared Imaging System (Li-Cor, Lincoln, NE), as described by the manufacturer.

### MTT Cytotoxicity Assay

*In vitro* HDACI cytotoxicities of pediatric AML cell lines were measured by using MTT (3-[4,5-dimethyl-thiazol-2-yl]-2,5-diphenyltetrazolium-bromide, Sigma) assays, as previously described [21]. Briefly, pediatric AML cell lines were cultured in 50 µl of RPMI 1640/20% dialyzed fetal bovine serum in 96-well plates. Cells were incubated at 37°C in the presence of varying concentrations of MS-275 (8 concentrations, range 0–2 µM), VPA (8 concentrations, range 0–9.6 mM), or SAHA (8 concentrations, range 0–4 µM). After 96 h, MTT was added to a final concentration of 1 mM. After 4.5 hours, formazan crystals were dissolved by the addition of 50 µl of 10% SDS in 10 mM HCl. Optical densities were measured with a visible microplate reader at 590 nm. IC_50_ values were calculated as drug concentrations necessary to inhibit 50% proliferation compared to untreated control cells. The data for the cell lines are presented as mean values ± standard errors from at least 3 independent experiments.

### shRNA Knockdown of HDACs in THP-1 cells

HDAC1, HDAC2, HDAC3, HDAC4, and HDAC6 shRNA lentivirus clones were purchased from the RNAi Consortium (Sigma-Aldrich) and used to infect THP-1 cells. After selection with puromycin, a pool of infected cells was expanded and tested for HDAC1, HDAC2, HDAC3, HDAC4, or HDAC6 expression by Western blotting (designated HDAC1-, HDAC2-, HDAC3-, HDAC4-, or HDAC6-shRNA). A pool of cells from the negative control transduction was used as the negative control (designated NTC-shRNA).

### Quantification of Gene Expression by Real-time RT-PCR

Total RNA isolation, cDNA preparation and purification were as previously reported [19], [20], [22]. Transcripts for *Bcl2L11* (encodes Bim) were quantitated using Taqman probes (Hs00197982_m1, Applied Biosystems Inc, Foster City, CA), and a LightCycler real-time PCR machine (Roche, Indianapolis, IN), based on the manufacturer's instructions. Real-time PCR results were expressed as mean values from 3 independent experiments using the same cDNA preparations and were normalized to GAPDH.

### Assessment of Baseline and Drug Induced Apoptosis

THP-1 cells were treated with HDACIs or cytarabine alone or in combination for up to 48 h. The cells were harvested, vigorously pipetted and triplicate samples taken to determine baseline and drug-induced apoptosis using the Apoptosis Annexin-V fluorescein isothiocyanate (FITC)/propidium iodide (PI) Kit (Beckman Coulter; Brea, CA), as previously described [19]. Apoptotic events were recorded as a combination of Annexin-V+/PI- (early apoptotic) and Annexin-V+/PI+ (late apoptotic/dead) events and results were expressed as percent of Annexin-V+ cells. Synergy was quantified using the cooperativity index (cooperativity index  =  sum of apoptosis of single agent treatment/apoptosis upon combined treatment). Cooperativity index <1,  = 1, or >1 is termed synergistic, additive, or antagonistic, respectively [23]. Percent changes of live cells relative to untreated controls were used to reflect inhibition on cell proliferation by the agents.

### Statistical Analysis

Differences in cell apoptosis between cytarabine- and HDACI-treated (individually or combined) and untreated cells or between HDAC shRNA knockdown clones and NTC cells were compared using the paired t-test. Statistical analyses were performed with GraphPad Prism 4.0.

## Results

### Expression Profiles of Classes I, II, and IV HDACs and HDACI Sensitivities in Pediatric AML Cell Lines

In our previous study, we demonstrated that VPA can enhance cytarabine-induced apoptosis in different subtypes of pediatric AML cells, including 4 cell lines (THP-1, Kasumi-1, CMS, and MV4-11) and 9 diagnostic blasts from children with *de novo* AML. Interestingly, Kasumi-1 and MV4-11 sublines were substantially more sensitive to VPA and showed greater responses to combined cytarabine/VPA, compared to THP-1 and CMS cells [7]. Our results strongly suggested that HDACs are promising therapeutic targets for treating pediatric AML with HDACIs, and that expression levels of certain HDACs could be responsible for the differential responses of the pediatric AML cells to VPA and combined VPA/cytarabine. However, the particular HDAC family members that impact cytarabine sensitivities have not been identified.

**Figure 1:**
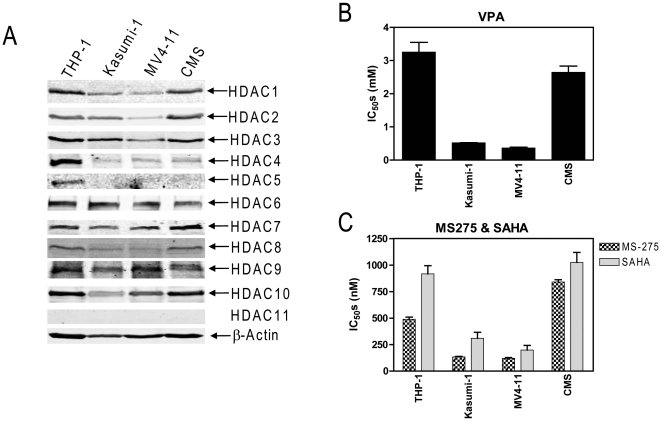
Expression profiles of classes I, II, and IV HDACs and sensitivities to HDACIs of pediatric AML cell lines. **Panel A**: Protein extracts from log phase THP-1, Kasumi-1, MV4-11, and CMS cells were subjected to Western blots probed by anti-HDAC or -β-actin antibodies. **Panels B&C**: THP-1, Kasumi-1, MV4-11, or CMS cells were cultured at 37°C for 96 h in complete medium with dialyzed fetal bovine serum in 96-well plates, with a range of concentrations of VPA, MS-275 or SAHA. Viable cell numbers were determined using the MTT reagent and a visible microplate reader. The IC_50_ values were calculated as the concentrations of drug necessary to inhibit 50% proliferation compared to control cells cultured in the absence of drugs. The data are presented as mean values ± standard errors from at least 3 independent experiments.

To begin to address this important question, we first determined the protein levels for class I, II, and IV HDACs in the 4 pediatric AML cell lines (THP-1, CMS, Kasumi-1, and MV4-11) used in our previous study [7]. All class I HDACs (1, 2, 3, and 8) and the majority of class II HDACs (4, 6, 7, 9, and 10) were detected in the cell lines, though the levels were somewhat variable. In contrast, HDAC5 was only detected in THP-1 cells and no detectable HDAC11 was found in any of the cell lines (Figure 1A).

Besides VPA (inhibits classes I and IIa HDACs, Figure 1B and reference 7), the 4 cell lines also showed differential sensitivities to MS-275 (a class I selective-HDACI) and SAHA (a pan-HDACI) [5], as determined by MTT assays (Figure 1C). Interestingly, the levels of class I HDACs positively correlated with the IC_50_s for the HDACIs and inversely correlated with the responses to combined VPA/cytarabine among the cell lines [7].

HDACs 5 and 11 are not likely to be involved in cytarabine sensitivities. While the remaining class II HDACs and any of the class I enzymes could be relevant to cytarabine antileukemic activities, based on the relationships between HDAC levels and responses to combined VPA/cytarabine [7], the impact of class I HDACs was most robust.

### Both Class I Selective- and pan-HDACIs Enhance Cytarabine-induced Apoptosis in Pediatric AML Cells

**Figure 2:**
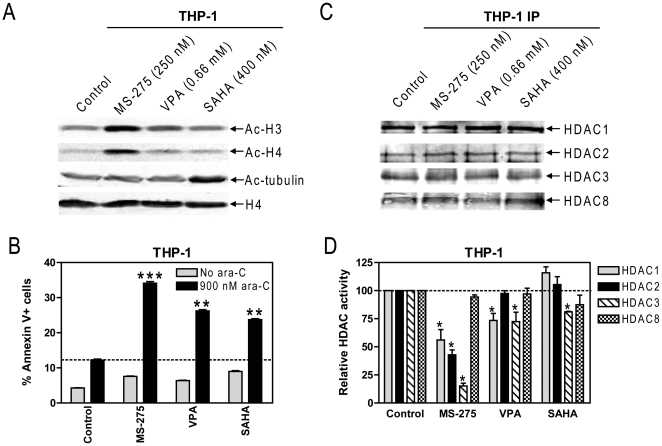
Effects of equal doses (IC_20_) of structurally-diverse HDACIs on acetylation of histones H3 and H4 and α-tubulin, and cytarabine-induced apoptosis in pediatric AML cells. **Panel A:** THP-1 cells were treated with equal doses (IC_20_s, determined by MTT assays) of MS-275, VPA or SAHA for 48 hrs. Acetylation of histones H3 and H4, and α-tubulin were determined by western blots probed by anti-ac-H3, -ac-H4, -ac-tubulin, or –H4 antibodies. **Panel B:** THP-1 cells were treated with cytarabine (900 nM, IC_20_) or equal doses of MS-275, VPA, or SAHA, alone or in combination for 48 hrs. Early and late apoptosis events were determined by annexin V/PI staining and flow cytometry analysis. **, *p*<0.005; *, *p*<0.05. **Panels C&D:** THP-1 cells were treated with equal doses (IC_20_s) of MS-275, VPA or SAHA for 48 hrs and protein extracts were subjected to immunoprecipitation with antibodies against class I HDACs, as described in the Materials and Methods. One half of the immunoprecipitated proteins was subjected to Western blots probed by anti-HDAC1, -HDAC2, -HDAC3, or -HDAC8 antibody (panel C), and the other half was used for measuring class I HDAC activities (panel D). The data are presented as means of three independent experiments normalized to the non-drug treatment controls.

To narrow down which HDACs are directly involved in cytarabine sensitivities in pediatric AMLs, we used equal doses (IC_20_s, determined by MTT assays) of the above HDACIs (MS-275, VPA, and SAHA) with diverse substrate specificities to treat THP-1 cells, characterized by high level expression of both class I and II HDACs. Interestingly, treatments of THP-1 cells with MS-275 resulted in the highest levels of acetylation of both histones H3 and H4, compared to VPA and SAHA (Figure 2A). In contrast, only treatment with SAHA resulted in hyperacetylation of α-tubulin, the substrate of HDAC6 (Figure 2A), suggesting the IC_20_ concentration for this drug was sufficient to inhibit class II HDACs in the cells. All three HDACIs enhanced cytarabine-induced apoptosis in THP-1 cells, with MS-275, VPA, and SAHA showing high, medium, and low levels, respectively, of synergistic enhancement response (cooperativity index <1.0, Figure 2B). These results imply that inhibition of class I HDACs can enhance cytarabine-induced apoptosis in pediatric AML cells. However, class II HDACs (e.g., HDAC6) are also implicated since SAHA was also effective. The variable enhancements of cytarabine-induced apoptosis by the HDACIs may be due to differential inhibition of individual HDACs or inhibition of different HDAC classes.

To test this, enzymatic activities of individual class I HDACs were measured post immunoprecipitation (IP) in THP-1 cells treated with IC_20_ concentrations of the HDACIs. HDACI treatments did not alter the levels of class I HDAC enzymes in the cells (Figure 2C). Interestingly, the HDACI treatments resulted in differential inhibition of class I HDAC enzymes. Thus, MS-275 treatment resulted in significant inhibition of HDACs 1, 2, and 3, VPA treatment resulted in significant inhibition of HDACs 1 and 3, while treatment with SAHA only inhibited HDAC3 (Figure 2D). It is interesting that the levels of apoptosis induced by the drug combinations in THP-1 cells (Figure 2B) inversely correlated with HDAC1 activities (Figure 2D), suggesting that HDAC1 may play a critical role in cytarabine-induced apoptosis. In contrast, none of the treatments resulted in significant inhibition of HDAC8 (Figure 2D), suggesting that HDAC8 is unlikely to be involved in cytarabine sensitivity. Together, our results suggest that the enhancement of cytarabine-induced apoptosis by MS-275 and VPA could be correlated with inhibition of HDACs 1, 2, and 3, while that by SAHA could be correlated with inhibition of HDAC3 and class II HDACs, at least HDAC6.

### shRNA Knockdown of HDACs 1 and 6 Augments Cytarabine-Induced Apoptosis in THP-1 Cells

**Figure 3:**
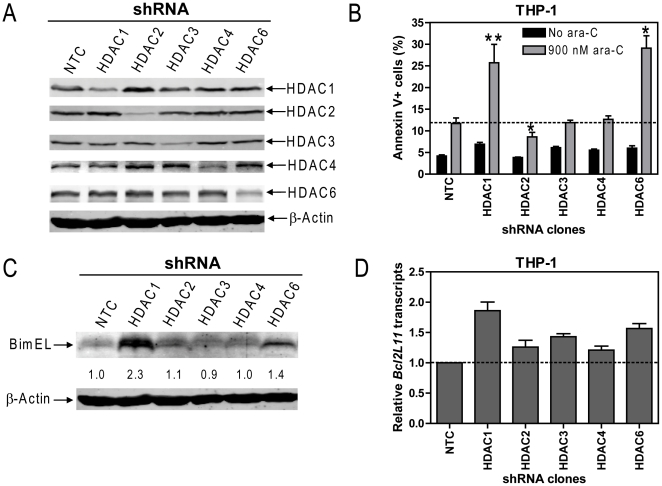
Effects of individual HDACs on cytarabine-induced apoptosis in THP-1 cells. **Panel A**: THP-1 cells were infected by HDAC1, HDAC2, HDAC3, HDAC4, HDAC6 or negative (NTC) control shRNA lentiviruses. After selection with puromycin, infected THP-1 cells were expanded and subjected to Western blotting and probing with anti-HDAC1, -HDAC2, -HDAC3, -HDAC4, -HDAC6, or -β-actin antibody. **Panel B**: shRNA stable clones were treated with 900 nM cytarabine for 48 h and early and late apoptosis events were measured using annexin V/PI staining and flow cytometry analysis. The horizontal line indicates the mean of cytarabine-induced apoptosis in the NTC-shRNA clone. **Panel C**: Whole cell lysates from shRNA stable clones were extracted and subjected to Western blotting probed by anti-Bim or -β-actin antibody. **Panel D**: Total RNAs were isolated from the shRNA stable clones and reverse transcribed to cDNA. Transcripts for *Bcl2L11* (encode Bim) were quantitated using a LightCycler real-time PCR machine. Real-time PCR results are presented as mean values from 3 independent experiments using the same cDNA preparation and normalized to GAPDH. **, *p*<0.005; *, *p*<0.01.

To further define the roles of the remainder of classes I and II HDACs in cytarabine sensitivities in pediatric AML, lentiviral shRNA knockdown of HDACs 1, 2, 3 (class I), 4 (representative of class IIa), and 6 (representative of class IIb) was performed in THP-1 cells. As shown in Figure 3A, all shRNAs resulted in markedly reduced (at least 50%) protein levels of the corresponding HDACs. Interestingly, down-regulation of only HDAC1 or HDAC6 resulted in significantly increased (∼2-fold) cytarabine-induced apoptosis compared to the NTC-shRNA cells. In contrast, down-regulation of HDACs 3 and 4 had no appreciable impact on cytarabine-induced apoptosis. Surprisingly, down-regulation of HDAC2 resulted in a slight (∼30%) yet significantly decreased apoptosis induced by cytarabine (Figure 3B). These results demonstrate that inhibition of HDACs 1 and 6 can significantly enhance cytarabine sensitivities in THP-1 cells, while inhibition of HDAC2 may negatively impact cytarabine sensitivity.

In our previous study, we found that VPA enhances cytarabine-induced apoptosis in pediatric AML cells accompanied by induction of the pro-apoptotic effector, Bim [7]. It is conceivable that Bim may also enable the enhancement of cytarabine-induced apoptosis of THP-1 cells resulting from down-regulation of HDACs 1 and 6. To test this possibility, real-time RT-PCR and Western blotting were performed in the shRNA stable clones. Interestingly, knock-down of HDACs 1 and 6 in the HDACs 1- and 6-shRNA clones was accompanied by substantially increased BimEL protein levels (2.3- and 1.4-fold, respectively) compared to the NTC-shRNA cells, while BimEL in the HDACs 2-, 3-, and 4- shRNA stable clones was largely unchanged (Figure 3C). The increased BimEL in the HDAC 1- and 6-shRNA stable clones was accompanied by substantially increased *Bcl2L11* transcripts (encode Bim) (1.9- and 1.6-fold, respectively), suggesting that a transcriptional mechanism may be responsible for the increased BimEL levels. Surprisingly, down-regulation of HDACs 2, 3, and 4 also resulted in increased levels for *Bcl2L11* transcripts accompanying unchanged BimEL protein (Figure 3D). These results indicate that the effects of HDACs 2, 3, and 4 on the expression of Bim must also involve post-transcriptional mechanisms. Together, our results suggest that both HDACs 1 and 6, but not HDACs 2, 3, and 4, are promising therapeutic targets for treating pediatric AML.

### HDACIs That Simultaneously Inhibit HDACs 1 and 6 Showed Greater Antileukemic Activities than HDACIs That Don't in Pediatric AML Cells

**Figure 4:**
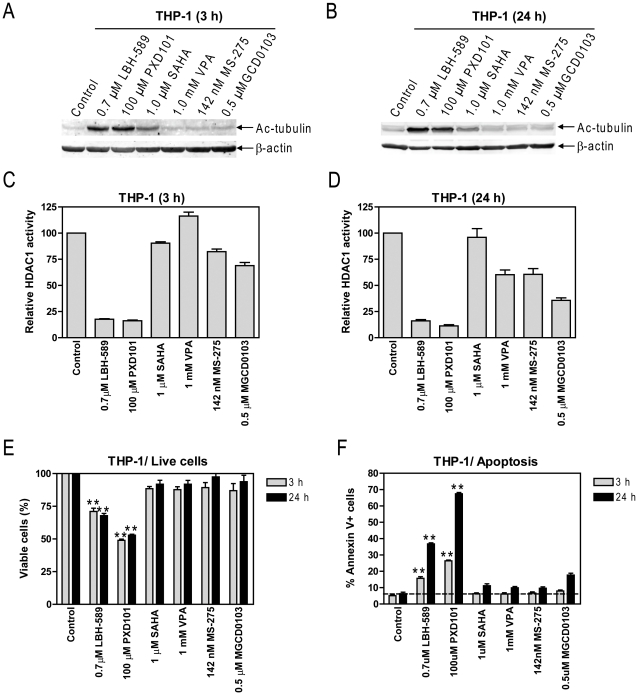
HDACIs That Simultaneously Inhibit HDACs 1 and 6 Showed Greater Antileukemic Activities than HDACIs that Don't at Cmax Concentrations. THP-1 cells were treated with LBH-589, PXD101, SAHA, VPA, MS-275 and MGCD0103 at C_max_ concentrations for 3 h and 24 h, respectively. The cells post 3 h treatments were washed three times with complete medium and divided into two halves. One half of the cells was resuspended in complete media and cultured for up to 24 h to determine the effects of the 3 h treatments on cell proliferation and apoptosis. The other half of the cells was used to prepare whole cell lysates. Whole cell lysates from the 3 h and 24 h treatments were extracted and subjected to Western blots probed by anti-ac-tubulin or –β-actin antibody (panels A&B), or subjected to HDAC1 enzymatic assays post IP as described in the Materials and Methods (Panels C&D). The effects of the 3 h and 24 h HDACI treatments on cell proliferation, as reflected by percent decrease of live cells relative to untreated cells (panel E), and apoptosis (panel F) were determined by flow cytometry analysis as described in the Materials and Methods.

Our results in pediatric AML cell lines suggest that simultaneous inhibition of HDACs 1 and 6 should result in better anti-leukemic effects than targeting HDAC1 or HDAC6 alone. To test this concept, THP-1 cells were treated for 3 h with HDACIs [LBH-589, PXD101, SAHA (pan-HDACIs), VPA (inhibits classes I and IIa HDACs), MS-275, MGCD0103 (class I selective-HDACIs)], all at *C_max_* concentrations from Phase I clinical trials (Table S1) [24], [25], [26], [27], [28], [29], [30], [31], [32], [33], [34], [35], [36], [37]. In order to establish the effects of these HDACIs on cell proliferation, THP-1 cells post 3 h treatments with the HDACIs were washed three times then resuspended in drug-free complete media and cultured for up to 24 h. The effects of the HDACIs on HDAC1 activity and acetylation of α-tubulin by HDAC6 were determined immediately following the 3 h treatments, whereas effects on cell proliferation and apoptosis were determined at 24 h. Consistent with previous reports [4], [5], treatments with LBH-589, PXD101, and SAHA, but not with the other HDACIs, resulted in hyperacetylation of α-tubulin, the substrate of HDAC6 (Figure 4A). IP followed by enzymatic assays revealed that both LBH-589 and PXD101 treatments resulted in the greatest inhibition of HDAC1 activities (>80% relative to control), compared to other HDACIs tested (Figure 4C). This was accompanied by significantly higher extents of proliferation inhibition (as reflected in percent decrease of live cells relative to untreated cells) and apoptosis (Figures 4E&4F). Essentially the same results were obtained in THP-1 cells when the HDACI treatments were extended to 24 h, though the levels of apoptosis induced by the drugs were substantially higher (Figures 4B, 4D, 4E&F). These results support the notion that simultaneous inhibition of HDACs 1 and 6 effects high levels of apoptosis in pediatric AML cells.

### DNA Damage and Bim Are Critical Determinants of HDACI-Induced Apoptosis in Pediatric AML Cells

**Figure 5:**
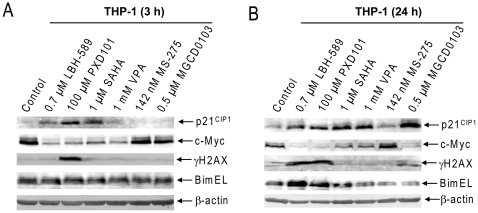
Induction of DNA Damage and Bim Is Critical for HDACI-Induced Apoptosis in Pediatric AML Cells. THP-1 cells were treated with the HDACIs at C_max_ concentrations for 3 (panel A) and 24 h (panel B), respectively. Whole cell lysates were extracted and subjected to Western blots probed by anti-p21, -c-Myc, -γH2AX, -Bim, or -β-actin antibody.

Efforts were undertaken to better understand the molecular mechanisms which underlie the anti-leukemic effects of the aforementioned HDACIs. Reports from our own group and others demonstrated that HDACIs can induce DNA damage [7], [38], which subsequently triggers apoptosis in leukemia cells. In addition, well documented HDAC targets, such as p21, c-Myc, and Bim, may also be relevant [4]. Interestingly, effects of the HDACIs on p21, c-Myc, and Bim expression, and in inducing DNA damage (as reflected in γH2AX) were both drug-dependent and time-dependent, as reflected in results at 3h and 24h (Figure 5). However, only induction of γH2AX and Bim paralleled the high levels of apoptosis upon treatment with LBH-589 and PXD101 (Figure 5). These results further support our previous conclusion that induction of DNA damage and Bim is critical for the anti-leukemic activities of HDACIs, whereas the roles of p21 and c-Myc remain to be established. It is also important to note that induction of Bim by HDACIs was apparently a late molecular event (i.e., compare results at 3 h and 24 h), consistent with our previous report [7].

### Simultaneous Inhibition of HDACs 1 and 6 Is Critical for Enhancing Cytarabine-Induced Apoptosis in Pediatric AML Cells

**Figure 6:**
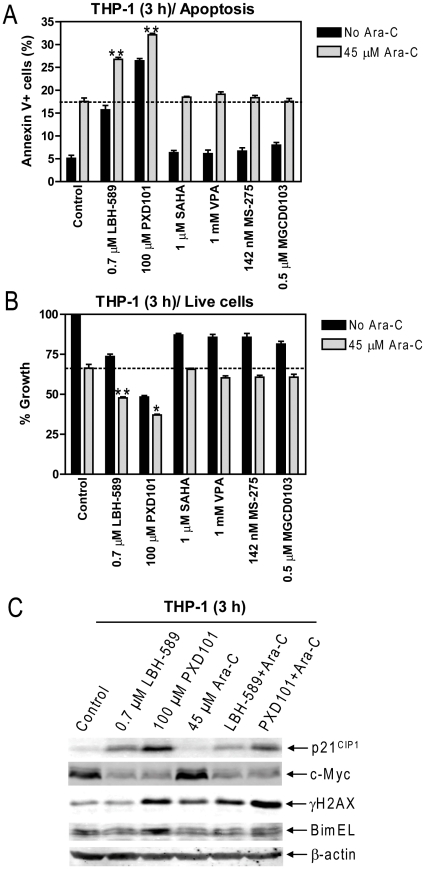
Simultaneous Inhibition of HDACs 1 and 6 Is Critical for Enhancing Cytarabine Induced-apoptosis in Pediatric AML Cells. THP-1 cells were treated with HDACIs or cytarabine, all at C_max_ concentrations, alone or combined for 3 h, washed three times with complete media and divided in two halves. One half of the cells was resuspended in drug-free complete media and cultured for up to 24 h. Effects of these treatments on apoptosis (panel A) and cell proliferation (as reflected in percent decrease of live cells relative to untreated cells, panel B) were determined by flow cytometry analysis as described in the Materials and Methods. ** indicates *p*<0.005. Whole cell lysates were extracted from the other half of the cells and subjected to Western blots probed by anti-p21, -c-Myc, -γH2AX, -Bim, or -β-actin antibody (panel C).

Our results from the shRNA knockdown experiments implied that simultaneous inhibition of HDACs 1 and 6 would result in greater enhancement of cytarabine sensitivities than targeting HDAC1 or 6 individually. To determine the impact of these HDACIs on cytarabine cytotoxicity and to mimic clinical treatment with cytarabine combined with these HDACIs, THP-1 cells were treated for 3 hours with the HDACIs with and without cytarabine, all at C_max_ concentrations (Table S1), analogous to experiments in Figure 4. The cells were washed three times then resuspended in complete media and cultured for up to 24 h. As expected, both LBH-589 and PXD101 significantly enhanced cytarabine-induced apoptosis and proliferation inhibition (as reflected in percent decrease of live cells compared to untreated cells) of THP-1 cells compared to the other HDACIs (Figures 6A&6B). This was accompanied by cooperative induction of DNA damage by the drug combinations, as reflected by the induction of γH2AX. In contrast, the drug combinations did not result in further changes for c-Myc (Figure 6C). These results further support the notion that HDACs 1 and 6 are indeed therapeutic targets in the treatment of pediatric AML and suggest that pan-HDACIs may exhibit optimal antileukemic activities at clinically achievable concentrations when combined with cytarabine compared to class I selective-HDACIs.

## Discussion

Leukemia is the most common form of childhood cancer and cancer is the leading cause of death from disease of American children. Hence, improving leukemia therapy is of utmost importance in pediatric health. This is particularly relevant to AML in which progress has lagged significantly in comparison to childhood acute lymphoblastic leukemia. Resistance to cytarabine-based chemotherapy is a major cause of treatment failure in this disease [2], [3]. Therefore, new therapies for children with AML must be developed.

HDACIs represent a promising new class of anti-cancer agents and can induce apoptosis in leukemia cells but not normal cells [4], [5]. In our previous study, we demonstrated that VPA, an anti-epileptic agent in both children and adults and a potent HDACI, synergistically enhanced cytarabine sensitivities in both pediatric AML cell lines and diagnostic blasts, suggesting that HDACs are promising therapeutic targets for pediatric AML [7]. However, individual HDAC family members that are involved in synergistic cytarabine response in the disease have not been identified.

This study was designed to begin to address this important question and to select the optimal HDACIs which show the greatest enhancement on cytarabine sensitivities in pediatric AML cells. Such information is mechanistically important and has significant clinical implications, as well. To begin to identify which HDAC isoforms are involved in cytarabine sensitivity, we examined the expression profiles of class I, II, and IV HDACs in 4 pediatric AML cell lines. Our results suggested that HDACs 5 and 11 are unlikely involved in cytarabine sensitivities due to the lack or marginal expression of these enzymes. Using THP-1 cells which express high levels of both classes I and II HDACs, we then used equal doses of three different HDACIs (MS-275, VPA, and SAHA) with different substrate specificities to further narrow down the HDAC isoforms likely to be involved in augmenting cytarabine sensitivity. Results from these studies suggested that HDAC8 is unlikely to be involved in cytarabine-induced apoptosis in THP-1 cells since none of the HDACI treatments resulted in significant enzyme inhibition, although they all enhanced cytarabine-induced apoptosis.

Results from our shRNA knockdown studies unequivocally demonstrated that inhibition of HDACs 1 and 6 was pivotal for sensitizing pediatric AML cells to cytarabine. This could, at least partly, be mediated by Bim, a BH3-only pro-apoptotic protein. Bim was classified as an “activator” in view of its purported ability to act directly and to activate Bax and Bak [39]. It has been well documented that Bim is critical for HDACI-induced apoptosis of both solid tumor and leukemia cells [40], [41]. Our previous study strongly suggested that Bim is also critical for cytarabine-induced apoptosis in pediatric AML cells [7]. Surprisingly, down-regulation of HDAC2 resulted in significantly decreased apoptosis induced by cytarabine, even though it was previously reported that down-regulation of HDAC2 is critical for inducing apoptosis in cancer cells [42]. In contrast, down-regulation of HDACs 3 and 4 had no effects on cytarabine-induced apoptosis in THP-1 cells. Together, our results strongly implicate both HDACs 1 and 6 as the most relevant therapeutic targets for treating pediatric AML with HDACIs and cytarabine. Studies are underway to determine the detailed molecular mechanisms responsible for the effects of HDACs 1, 2, and 6 on cytarabine sensitivities in the disease.

It has been a long-standing debate as to whether isoform specific- or pan-HDACIs result in better anti-cancer activities [43], [44]. The perception is that isoform-specific HDACIs may offer clear therapeutic advantages over non-specific classical HDACIs. Specifically, the premise is that the greater specificity will involve the modulation of a smaller number of disease-focused genes with a reduced toxicity profile [44]. However, recent microarray studies suggested that the pleiotropic antiproliferative and apoptotic effects of the broad-spectrum HDACIs may be more beneficial than an isoform-specific drug [16], [45]. Our results from shRNA knockdown studies strongly favor the latter opinion, at least in pediatric AML, since both HDACs 1 and 6 appear to be critical factors in determining cytarabine sensitivities in the disease. This was further supported by our *in vitro* treatments of pediatric AML cells with both class I selective- and pan-HDACIs. At clinically achievable concentrations, only the drugs (LBH-589 and PXD101) which simultaneously inhibited both HDACs 1 and 6 showed the best antileukemic activities and significantly enhanced cytarabine-induced apoptosis. Again, our mechanistic studies suggest that induction of DNA damage and Bim is critical for the activities of LBH-589 and PXD101 and their combinations with cytarabine.

Altogether, our results not only confirmed that HDACs are promising therapeutic targets for pediatric AML, but also identified HDACs 1 and 6 as the most relevant drug targets. Accordingly, treating pediatric patients with pan-HDACIs may be more beneficial than HDAC isoform-specific drugs. Our study provides compelling rationale for the combination of cytarabine and HDACIs in pediatric AML clinical trials. It also provides a strong molecular basis for selecting the optimal HDACIs to combine with cytarabine. Since many biological features of AML are shared by adults and children, our results should also apply to the treatment of adult AML patients, as well. It is extremely important to note that we used C_max_ concentrations for the HDACIs to combine with cytarabine to prove the concept. However, C_max_ or the maximally tolerated doses of these HDACIs may not be the optimal doses for combination therapy with cytarabine. Detailed preclinical studies will be needed to establish the optimal scheduling and dosing for the combinational therapies involving cytarabine plus LBH-589 or PXD101 for treating pediatric AML.

## Supporting Information

Table S1 **C_max_ and t_1/2_ values of HDACIs determined by phase I clinical trials**
(DOC)
