# Supplementary material for: Inhibition of Histone Deacetylases 1 and 6 Enhances Cytarabine-Induced Apoptosis in Pediatric Acute Myeloid Leukemia Cells
Source: PLoS One. 2011 Feb 16;6(2):e17138. doi: 10.1371/journal.pone.0017138 (PMC3040224; doi:10.1371/journal.pone.0017138)
Supplement: Table S1 — Cmax and t1/2 values of HDACIs determined by phase I clinical trials (DOC) [file pone.0017138.s001.doc]

**Table S1. C_max_ and t_1/2_ values of HDACIs determined by phase I clinical trials**

| **HDACIs** | **C_max_** | **t_1/2_** | **Reference** |
| --- | --- | --- | --- |
| LBH-589 | 0.7 µM | 9.0 hrs | 29 |
| PXD101 | 100.0 µM | 0.3-1.3 hrs | 35 |
| SAHA | 1.0 µM | 1.0-1.9 hrs | 26,28 |
| VPA | 1.0 mM | 6.0 hrs | 36 |
| MS-275 | 83-142 nM | 33.0 hrs | 30-33 |
| MGCD0103 | 0.5 µM | 9.5 hrs | 27,34 |
| Ara-C | 44.6-94.0 µM | 0.44-2.8 hrs | 37-39 |
